# Volumetric MRI is a promising outcome measure of muscle reinnervation

**DOI:** 10.1038/s41598-021-01342-y

**Published:** 2021-11-17

**Authors:** Matthew Wilcox, Liane Dos Santos Canas, Rikin Hargunani, Tom Tidswell, Hazel Brown, Marc Modat, James B. Phillips, Sebastien Ourselin, Tom Quick

**Affiliations:** 1 Peripheral Nerve Injury Research Unit, Royal National Orthopaedic Hospital, Stanmore, UK; 2 UCL Centre for Nerve Engineering, University College London, London, UK; 3 Department of Pharmacology, UCL School of Pharmacy, University College London, London, UK; 4 Biomedical Engineering and Imaging Sciences, King’s College London, London, UK; 5 Department of Radiology, Royal National Orthopaedic Hospital, Stanmore, UK; 6 Department of Clinical Neurophysiology, Royal Free Hospital, London, UK; 7 University College London Medical School, London, UK

**Keywords:** Somatic system, Peripheral nervous system, Regeneration and repair in the nervous system, Biomarkers, Neurology, Neurological disorders

## Abstract

The development of outcome measures that can track the recovery of reinnervated muscle would benefit the clinical investigation of new therapies which hope to enhance peripheral nerve repair. The primary objective of this study was to assess the validity of volumetric Magnetic Resonance Imaging (MRI) as an outcome measure of muscle reinnervation by testing its reproducibility, responsiveness and relationship with clinical indices of muscular function. Over a 3-year period 25 patients who underwent nerve transfer to reinnervate elbow flexor muscles were assessed using intramuscular electromyography (EMG) and MRI (median post-operative assessment time of 258 days, ranging from 86 days pre-operatively to 1698 days post- operatively). Muscle power (Medical Research Council (MRC) grade) and Stanmore Percentage of Normal Elbow Assessment (SPONEA) assessment was also recorded for all patients. Sub-analysis of peak volitional force (PVF), muscular fatigue and co-contraction was performed in those patients with MRC > 3. The responsiveness of each parameter was compared using Pearson or Spearman correlation. A Hierarchical Gaussian Process (HGP) was implemented to determine the ability of volumetric MRI measurements to predict the recovery of muscular function. Reinnervated muscle volume per unit Body Mass Index (BMI) demonstrated good responsiveness (R^2^ = 0.73, *p* < 0.001). Using the temporal and muscle volume per unit BMI data, a HGP model was able to predict MRC grade and SPONEA with a mean absolute error (MAE) of 0.73 and 1.7 respectively. Muscle volume per unit BMI demonstrated moderate to good positive correlations with patient reported impairments of reinnervated muscle; co- contraction (R^2^ = 0.63, *p* = 0.02) and muscle fatigue (R^2^ = 0.64, *p* = 0.04). In summary, volumetric MRI analysis of reinnervated muscle is highly reproducible, responsive to post-operative time and demonstrates correlation with clinical indices of muscle function. This encourages the view that volumetric MRI is a promising outcome measure for muscle reinnervation which will drive advancements in motor recovery therapy.

## Introduction

Peripheral nerve injuries (PNI) occur in around 2% of all trauma cases and represent a significant global health challenge^1–4^. Increasing identification of regenerative therapies which enhance nerve regeneration in preclinical models drives a concomitant need for outcome measures of reinnervated muscle^5,6^. Ideal outcome measures are reproducible, responsive to the biological process of muscle reinnervation, correlate with clinical measurements of muscular function and are non-invasive to perform^7^.

Neurophysiological assessment is widely used to diagnose nerve injuries and monitor recovery of reinnervated muscle^8–11^. Compound Muscle Action Potential (CMAP) readings have been used as outcome measures to track disease progression in neuromusucular pathologies^12–15^. However, it is often not possible to achieve reproducible and accurate recordings in proximal muscles such as the elbow flexors. This is because percutaneous stimulation of the entire motor point of proximal nerve trunks such as the musculocutaneous nerve trunk is challenging^15^. Intramuscular quantitative electromyography (EMG) including Motor Unit Action Potential (MUAP) analysis and/or semi-quantitative measurements of spontaneous activity may circumvent this issue^9^. However, this method can be uncomfortable for the patient and depends on their co-operation. Further, in longitudinal studies it can be difficult for the examiner to relocate the same topographical area for assessment of changes in these neurophysiological parameters.

Developing improved outcome measures is important to track change in clinical trials and to identify cases where surgical intervention may offer clinical benefit. However, this is challenging in the context of human muscle reinnervation. The rate of human nerve regeneration is slow, around 1 mm/day^16^, therefore small incremental changes over time may be masked by environmental factors and/or measurement variation. Furthermore, the intricate and often varied anatomy in addition to diverse range of injuries make PNI a heterogeneous pathology to study. The nerve transfer to restore elbow function (the Oberlin’s procedure) has been identified as a surgical scenario of human muscle reinnervation which circumvents many of these issues^17–19^. In this procedure, the surgeon creates a controlled injury to an uninjured fascicle of the ulnar nerve and redirects these axons to grow into the denervated musculocutaneous nerve to the elbow flexor muscles.

In animal models, muscle wet weight is often utilised as an outcome measure by researchers to establish the efficacy of therapies following nerve repair and to predict functional recovery^5,20^. Unfortunately, measurement of muscle wet weight in humans is not feasible. However, the advent of quantitative Magnetic Resonance Imaging (MRI) techniques has enabled researchers to non-invasively measure muscle volume. Assessment of the reproducibility, responsiveness and correlation with clinical metrics of muscular function would represent a key step towards validating volumetric MRI as an outcome measure of muscle reinnervation.

The clinical assessment of reinnervated muscular function is complex. Established objective assessments of muscular function are often restricted to the measurement of peak volitional force (PVF) through the Medical Research Council (MRC) grading system of muscle power and/or handheld dynamometry (HHD) measurements^21,22^. However, recent studies of patient reported impairments have identified an earlier onset of muscular fatigue, pathological co-contraction, altered proprioception and muscle pain as central themes of chronically reinnervated muscle (> 1 year following reconstructive nerve surgery)^18,23^.

The importance of the use of patient reported outcomes (PROs) in combination with clinical reported outcomes within both research and the clinical setting is well recognized^24^. The authors (HB and TQ) developed the Stanmore Percentage of Normal Elbow Assessment (SPONEA)^25^ as a PRO of muscle reinnervation by adapting a pre-existing assessment of shoulder function; the Stanmore Percentage of Normal Shoulder Assessment (SPONSA)^26^. This tool provides a patient reported assessment of strength, range of movement, pain and the functional ability of the elbow joint following nerve transfer to reinnervate elbow flexor muscles. Ascertaining the relationship of these objective and subjective clinical assessments with volumetric MRI assessment of reinnervated muscles would represent a key step towards establishing priorities for motor recovery therapy.

The aim of this study was to establish the validity of volumetric MRI as an outcome measure of muscle reinnervation by testing its reproducibility, responsiveness and relationship with clinical indices of muscle function. The validity of volumetric MRI was compared to conventional semi-quantitative neurophysiological markers of muscle reinnervation including spontaneous activity and MUAP analysis. In order to achieve this, patients who underwent nerve transfer to reanimate elbow flexion were followed-up at a range of pre- and post-operative time points for MRI, neurophysiological and clinical assessment of their reinnervated elbow flexor muscles.

## Results

### Clinical features

Twenty-five patients, 23 males and two females, were included in this study with a median age of 34.5 years (ranging from 23 to 66 years). There were ten right-sided and 15 left-sided brachial plexus injuries; 14 and 11 of which were on the dominant and non-dominant side respectively. Twenty-one of the injuries were due to motorbike accidents, two were following bicycle accidents, one following a car accident and one due to a skiing accident. Intra-operatively, 22 patients were found to have C5/6 avulsion, one had a C5-8 avulsion, one had a C5-7 avulsion and one had axonotmesis of biceps branch of musculocutaneous nerve (Additional file 1). Six healthy male volunteers with a median age of 34.5 (ranging from 24 to 52) underwent MRI scans of their elbow flexor muscles on their dominant (four were right-handed and two were left-handed) side for comparison to nerve injured arms.

### Neurophysiological investigation

A weak to moderate positive linear correlation was found between semi-quantitative assessment of spontaneous activity and post-operative time (R^2^ = 0.43, *p* = 0.03) (Supplementary Material Fig. 2A). Measurement of the magnitude of motor units (MUs) demonstrated relatively poor responsiveness to post-operative time (R^2^ = 0.36, *p* = 0.002) (Supplementary Material Fig. 2B).

### Scan-rescan, inter- and intra-investigator reproducubility of the MRI segmentation protocol

All reproducibility measurements were carried out on healthy controls. T1-w scans yielded the highest scan-rescan (0.95 (0.50–0.93)), inter- (0.98 (0.98–1.00)) and intra-investigator (0.99 (0.98–1.00)) reproducibility of volumetric measurements out of all the imaging sequences employed in this study (Supplementary Material Fig. 3). Therefore, T1-w images were used for all volumetric assessments.

### Volumetric analysis

**Figure 1:**
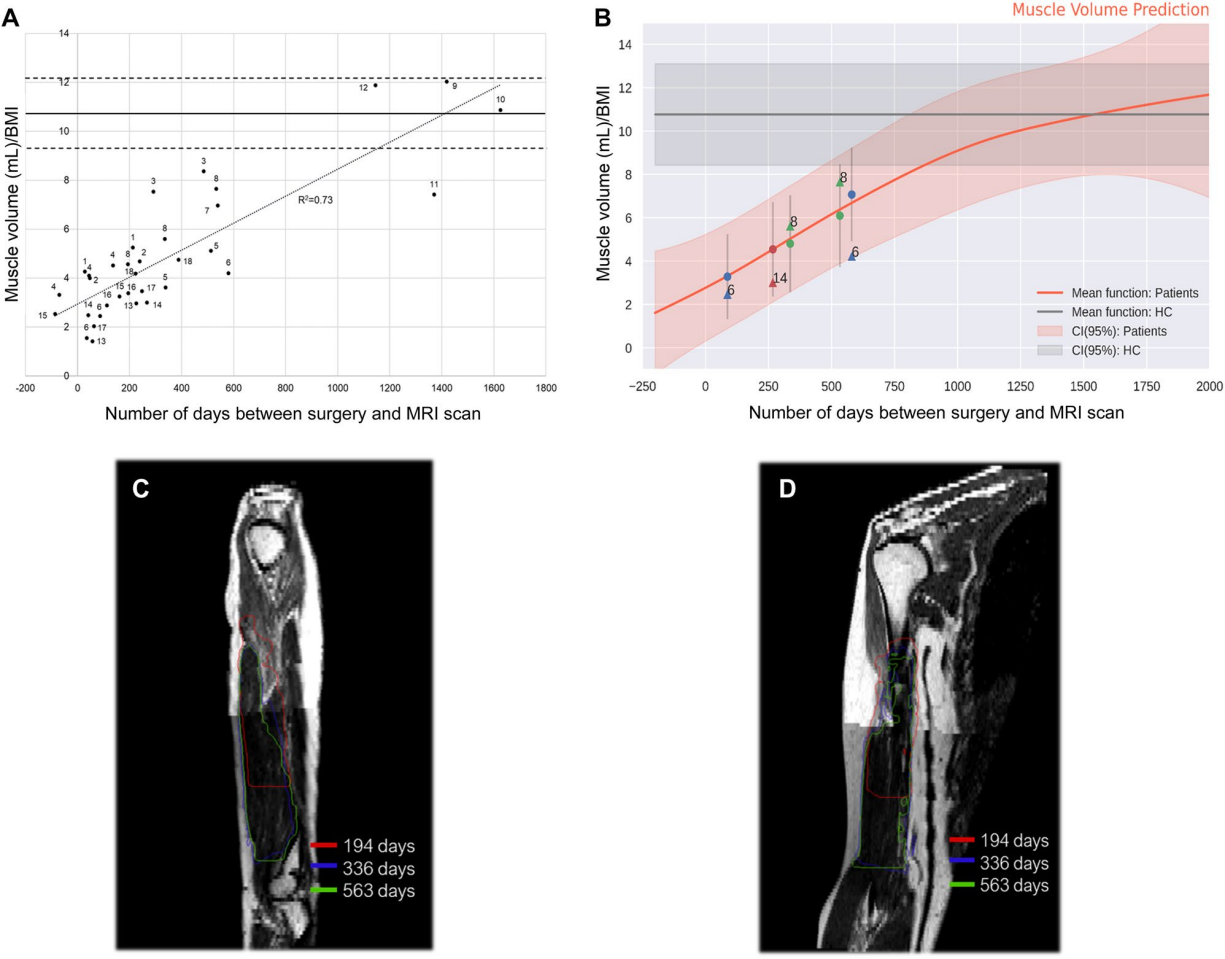
Changes in elbow flexor muscle volume pre- and post-nerve transfer. The numbers attached to the data points in (**A, B)** are in reference to the case numbers provided in Additional file 1. (**A)** Quantification of elbow flexor muscle volume at pre- and post-nerve transfer time points. The solid black line represents the mean uninjured elbow flexor muscle volume (n = 6) and the dashed black lines represent ± one standard deviation from the mean. (**B)** Application of a Hierarchical Gaussian Process model to the data presented in (**A)**. The solid black line represents the mean muscle volume/BMI of uninjured elbow flexor muscles (n = 6) and the shaded area represents one standard deviation from the mean. The circular data points represent the predicted values on the triangular points denote the actual data points. (**C–E)** Represents deformable registration of elbow flexor muscle segmentations from case number 8 (Additional file 1) 194, 336 and 553 days post-nerve transfer. (**C)** Sagittal plane. **(D)** Frontal plane. Supplementary Video 1 shows the 3D reconstruction video of reinnervated elbow flexor muscles of **C** and **D**.

The median Body Mass Index (BMI) of the healthy controls was 25 (ranging from 22 to 29). The mean muscle volume per unit BMI of uninjured elbow flexor muscles was 10.76 mL per unit BMI (± 1.42) (Fig. 1A,B). The volume per unit BMI of elbow flexor muscles demonstrated a strong positive linear correlation with pre- and post-operative time points (R^2^ = 0.73, *p* < 0.001) (Fig. 1A). A Hierarchical Gaussian Process (HGP) model was implemented to predict changes in this biomarker at pre- and post-operative time points (Fig. 1B). The HGP model was able to predict muscle volume per unit BMI at a given time point with a mean absolute error (MAE) of 0.9 mL per unit BMI and a mean Log likelihood of 0.15 (Supplementary Material Fig. 4). Visual representations of reinnervated elbow flexor muscle volume measurements from case number 8 (Additional file 1) post-operatively are shown in Fig. 1C–E.

### Objective assessments of muscular function

#### MRC grading

**Figure 2:**
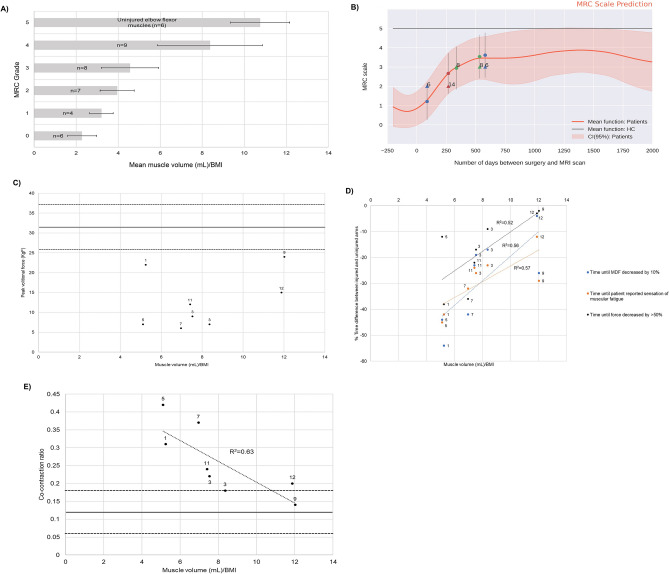
Relationship between muscle volume per unit BMI and objective measurements of muscular function. The error bars in (**A)** represent one standard deviation from the mean. In (**B–E)** case numbers are attached in reference to Additional file 1 which also provides details pertaining to the time interval between injury and surgery as well as surgery and clinical assessments. In (**C, E),** the solid black line represents the mean PVF and co-contraction ratio obtained from the uninjured contralateral arms respectively whilst the dashed lines represent one standard deviation from the mean. (**A)** An MRC grade of 0, 1, 2, 3 and 4 was associated with a mean reinnervated muscle volume per unit BMI of 2.28 (± 0.7), 3.20 (± 0.56), 3.96 (± 0.81), 4.57 (± 1.36) and 8.37 (± 2.49) respectively. Uninjured muscles (MRC grade 5) had a mean muscle volume per unit BMI of 10.76 (± 1.42). There was a statistically significant difference in muscle volume per unit BMI between MRC grade 4 and 0, 5 and 0, 4 and 1, 5 and 1, 4 and 2, 5 and 2, 4 and 3 as well as 5 and 3 as assessed by a one-way analysis of variance (ANOVA) and Bonferroni test. (**B)** Application of a Hierarchical Gaussian Process model to the data presented in (**A).** The circular data points represent the predicted values on the triangular points denote the actual data points. (**C)** The relationship between PVF and muscle volume (mL) per unit BMI. (**D)** The relationship between sEMG, force and subjective measurements of muscular fatigue and muscle volume (mL) per unit BMI. **(E)** The relationship between co-contraction and muscle volume (mL) per unit BMI.

Volumetric measurements directly correlated with function. An MRC grade of 0, 1, 2, 3 and 4 was associated with a mean reinnervated muscle volume per unit BMI of 2.28 (±0.7), 3.20 (±0.56), 3.96 (±0.81), 4.57 (±1.36) and 8.37 (±2.49) respectively (Fig. 2A). Uninjured muscles (MRC grade 5) had a mean muscle volume per unit BMI of 10.76 ( ±1.42) (Fig. 2A). There was a statistically significant difference (*p*<0.001) in muscle volume per unit BMI between MRC grade four and zero, five and zero, four and one, five and one, four and two, five and two, four and three as well as five and three (Fig. 2A). Using the muscle volume per unit BMI and temporal measurements from Fig. 1B, the HGP model was used to infer objective (MRC grading) (Fig. 2B). The HGP model was able to predict MRC grade with an MAE of 0.73 and a mean Log likelihood of 0.28 (Supplementary Material Fig. 4). This means that the HGP model was able to predict within ±1 MRC grade.

#### PVF measurements

The population mean force generated by reinnervated elbow muscles was 12.55KgF ( ±6.75) compared to 31.51KgF ( ±5.67) for uninjured arms. This was a statistically significant differential (*p*<0.0001). No significant correlation between the recovery of PVF and muscle volume per unit BMI was found (Fig. 2C).

#### Muscular fatigue evaluation

##### sEMG

The mean Median Frequency (MDF) of injured elbow flexor muscles across the first 10 seconds of the sustained isometric contraction was 62.90Hz (±5.89) compared to 73.13Hz (±4.62) for uninjured arms. This was a statistically significant differential (p=0.0017). The population mean time taken for this index MDF value to fall by 10% was 22.84 seconds (±12.41) for reinnervated muscles compared to 32.10 seconds (±9.46) for uninjured arms. This was not a statistically significant difference (*p*=0.1154). Overall, the percentage difference in time taken for MDF to fall by 10% between reinnervated and uninjured arms demonstrated a significant positive linear correlation with muscle volume per unit BMI measurements (R^2^=0.56, *p*=0.03) (Fig. 2D).

##### Force

The mean time until there was a 50% drop in force during the sustained isometric contraction was 32.21 seconds (±7.14) for reinnervated elbow flexor muscles and 39.12 seconds (±8.26) for uninjured muscles representing a non-statistically significant difference (*p*=0.8312). The percentage difference in time until there was a 50% reduction in force output during the isometric contraction between reinnervated and uninjured arms demonstrated a significant positive linear correlation with the muscle volume per unit BMI readings (R^2^=0.52, *p*=0.04) (Fig. 2D).

##### Subjective

The mean time interval between the start of the isometric contraction and the onset of subjective muscular fatigue was 29.06 seconds (±6.32) for reinnervated muscles compared to 40.87 seconds (±13.62) for uninjured arms. This was a statistically significant differential (*p*=0.0431). The percentage difference in time taken for the onset of subjective muscular fatigue between reinnervated and uninjured arms demonstrated a significant positive linear correlation with muscle volume per unit BMI measurements (R^2^=0.57, *p*=0.04) (Fig. 2D).

#### Co-contraction measurements

The population mean co-contraction ratio for the uninjured contralateral arms was 0.12 (± 0.06) compared to 0.26 (±0.09) within reinnervated arms; a statistically significant differential (*p*=0.0026). Focusing on reinnervated arms, co-contraction ratio demonstrated a moderate to good significant negative linear correlation with muscle volume per unit BMI (R^2^=0.63, *p*=0.02) recovering to similar levels as uninjured muscle (Fig. 2E).

### Subjective assessment of muscular function

**Figure 3:**
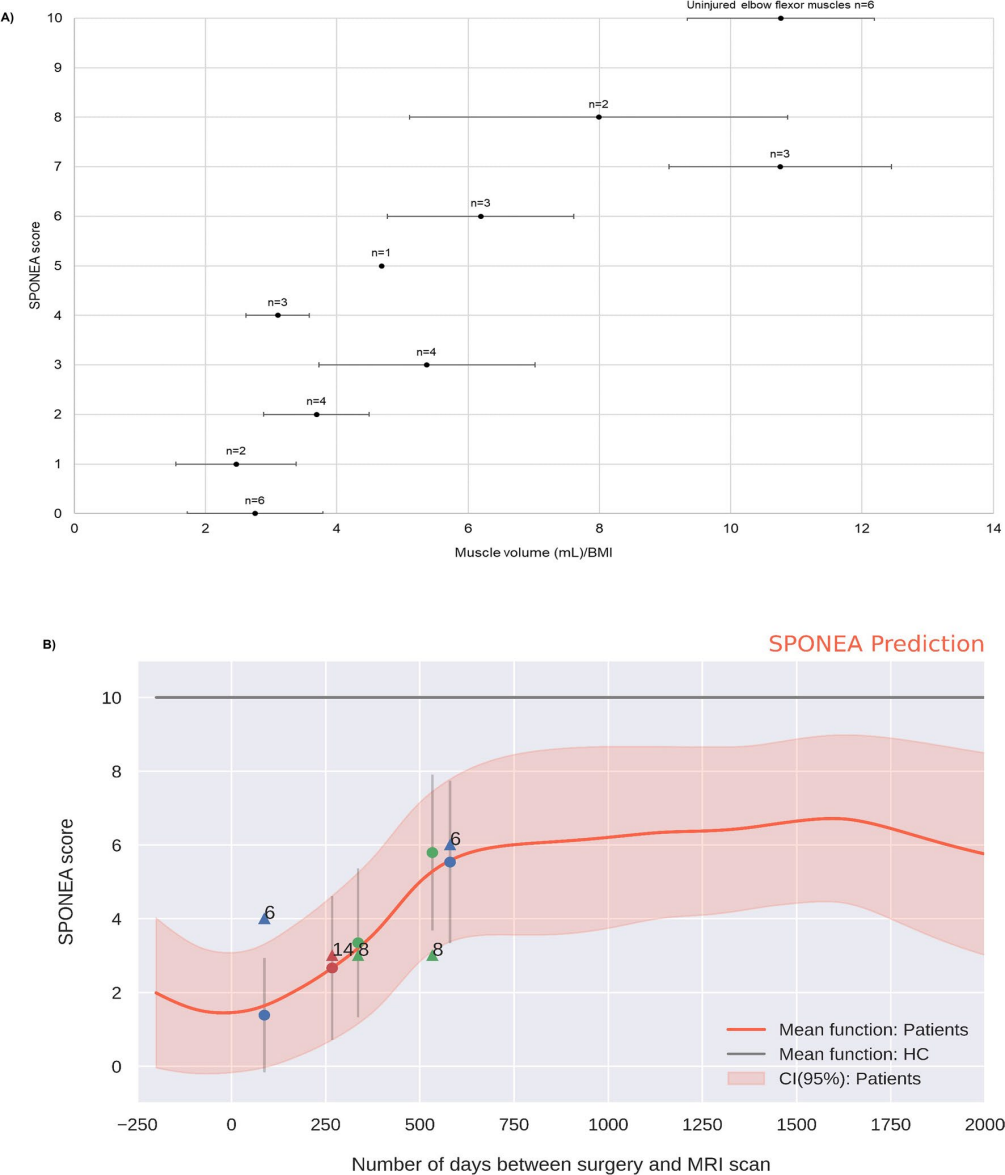
Relationship between muscle volume per unit BMI and SPONEA. **(A)** Relationship of the mean muscle volume per unit BMI with the SPONEA scale. The error bars represent one standard deviation. (**B)** Application of the HGP to the data presented in (**A)**. The numbers attached to the data points are in reference to the case numbers provided in Additional file 1. The circular data points represent the predicted values on the triangular points denote the actual data points.

SPONEA demonstrated a strong positive linear correlation with muscle volume per unit BMI (R^2^ = 0.45, *p* < 0.001) (Fig. 3A). Using the muscle volume per unit BMI and temporal data from Fig. 1B, the HGP model was implemented to infer the subjective recovery of elbow flexion at different time points post-operatively (Fig. 3B). The MAE of 1.7 and mean Log likelihood of 0.15 suggests that the HGP model can predict SPONEA within two points (Supplementary Material Fig. 4).

## Discussion

A major barrier to the clinical translation of new therapies to enhance peripheral nerve repair is the absence of outcome measures that are responsive and demonstrate correlation with clinical metrics of reinnervated muscular function^5^. To address this issue, a study was undertaken to the determine the extent to which muscle volume relates to objective and subjective assessments of reinnervated muscle.

The current clinical standard for monitoring muscle reinnervation is intramuscular EMG^9^. However, the findings suggest that intramuscular EMG parameters have low responsiveness warranting research into improved outcome measures of muscle reinnervation.

The findings show that the segmentation protocol deployed in this study to measure elbow flexor muscle volume demonstrated excellent inter- (ICC = 0.92 (0.80–0.97)) and intra- (ICC = 0.99 (0.97–1.00)) investigator reproducibility^7^. This concurs with other studies that have used quantitative MRI techniques to measure biological changes associated with neuromuscular pathologies such as Charcot-Marie-Tooth disease (CMT)^27–30^. This is important because this finding suggests volumetric MRI may be a valuable tool in multi-centre clinical trials.

The development of responsive outcome measures is critical in the context of clinical nerve repair research. Successful clinical translation of novel regenerative therapies will likely depend on the ability of outcome measures to capture small incremental changes over time^31^. Conventional clinical metrics such as MRC grading are not sufficiently sensitive to the biological process of muscle reinnervation and therefore limit the design of clinical trials of therapies which hope to enhance nerve repair^31–33^.

Quantification of reinnervated elbow flexor muscle volume per unit BMI demonstrated improved responsiveness when compared with conventional neurophysiological indicators of muscle reinnervation. This concurs with findings in animal models of nerve repair which demonstrate that measurements of muscle mass are sensitive to the biological process of muscle reinnervation^34^. Controlled animal models of muscle reinnervation have shown that therapies such as growth hormone^35,36^ lead to an approximately 20% higher recovery of muscle mass when compared to control. Application of this finding to the HGP modelling of the volumetric data presented in this study can inform power calculations for future clinical trials; a study which hopes to capture a 20% improvement in muscle volume with 80% power and 0.05 alpha at 180 days post-nerve transfer would require 55 patients each in the control and experimental group. Other factors such as resistance training which may form a component of physical rehabilitation following surgical nerve repair are known to correlate with volumetric assessments^37^. Measurements of muscle mass and volume may provide improved correlation with clinical indices of muscle reinnervation compared with neurophysiological parameters since they provide a more direct assessment of the functional recovery of MUs rather than changes in the electrical properties of MUs as assessed by neurophysiological indices^9^.

Delineating the relationship of muscle volume per unit BMI with objective and subjective clinical assessments of muscular function will allow researchers to define primary endpoints in human trials.

Reinnervated muscles assigned MRC grade four demonstrated approximately double the range of muscle volume per unit BMI readings when compared to lower MRC grades. This finding reflects the well documented limitations of the MRC grading system when monitoring the functional recovery of reinnervated muscle; over 96% of MRC evaluations are determined as MRC grade 4^32^. However, patient reported impairments of muscle reinnervation such as muscular fatigue and co-contraction demonstrated improved correlation with muscle reinnervation. This suggests that the clinical assessment of reinnervated muscular function should go beyond PVF measurement alone to better embody the recovery of afferent muscular function.

SPONEA was found to be responsive to volumetric measurements. Recent studies that have adopted percentage of normal assessment tools to appraise subjective muscular function in neuromuscular pathologies have reported similar findings^26,30,38–40^. This encourages the view that percentage of normal assessments should be more widely adopted by clinicians as a subjective evaluation of reinnervated muscular function.

The findings of this study must be interpreted in light of its limitations. The effect of the time interval between injury and surgery was not considered. An incremental increase in time between injury and surgery leads to a tissue microenvironment that becomes increasingly antagonistic to axonal regeneration and muscle reinnervation^41–43^. Future studies should also consider obtaining baseline, pre-operative volumetric measurements to better understand the rate of muscle volume change post-operatively. In addition, the data would have benefited from standardised neurophysiological and MRI follow-up time points with a larger cohort of patients. This will help the mathematical modelling data presented in this paper better predict functional recovery before conventional markers of muscle reinnervation can determine whether nerve repair has been successful or not. By extension, this will allow earlier clinical interventions to be made before the tissue microenvironment becomes antagonistic to muscle reinnervation. This was challenging in the present study since EMG and MRI equipment was housed at different clinical locations meaning that patients had to attend multiple research appointments which may have deterred patients. Since this study was conducted at a national referral centre, patients often had to travel from far afield which may have further reduced follow-up.

Animal models have shown that females appear to exhibit faster rates of nerve repair compared to males^44^. Studies which address whether this translates into differential rates of volumetric recovery in humans will help characterise the natural history of nerve repair and inform the design of clinical trials. Focusing on the HHD measurements, future studies may wish to consider modifying functional assessments such that the participant performs an isometric contraction against a fixed object rather than an investigator. This will ensure that these functional assessments measure the participant’s muscular function in isolation rather than the ability of the investigator to endure the efforts of the participant. Additionally, brachial plexus injury is a relatively rare pathology which restricted the recruitment of a larger cohort of patients. There was also a shortage of data between approximately 600 and 1100 days post-operatively. In agreement with current guidelines, patients were evaluated for discharge from clinic around 2-years following nerve transfer affording challenges to the long term follow-up of these patients^45^. This is characteristic of studies involving trauma with follow-up rates reported to be as low as 2%^46^.

In summary, volumetric MRI demonstrates reproducibility, temporal responsiveness and correlation with clinical assessments of muscular function. This suggests volumetric MRI is an excellent candidate as an outcome measure of muscle reinnervation. Future work should establish whether volumetric MRI can capture a meaningful clinical response in clinical trials of therapies which hope to enhance human nerve repair.

## Methods

This study received full ethical approval from REC reference 17/YH/0413, IRAS ID 235012, REC reference: 16/LO/0623; IRAS ID: 202847 and REC reference 17/WM/0438, IRAS ID 231428.

### Surgical procedure

**Figure 4:**
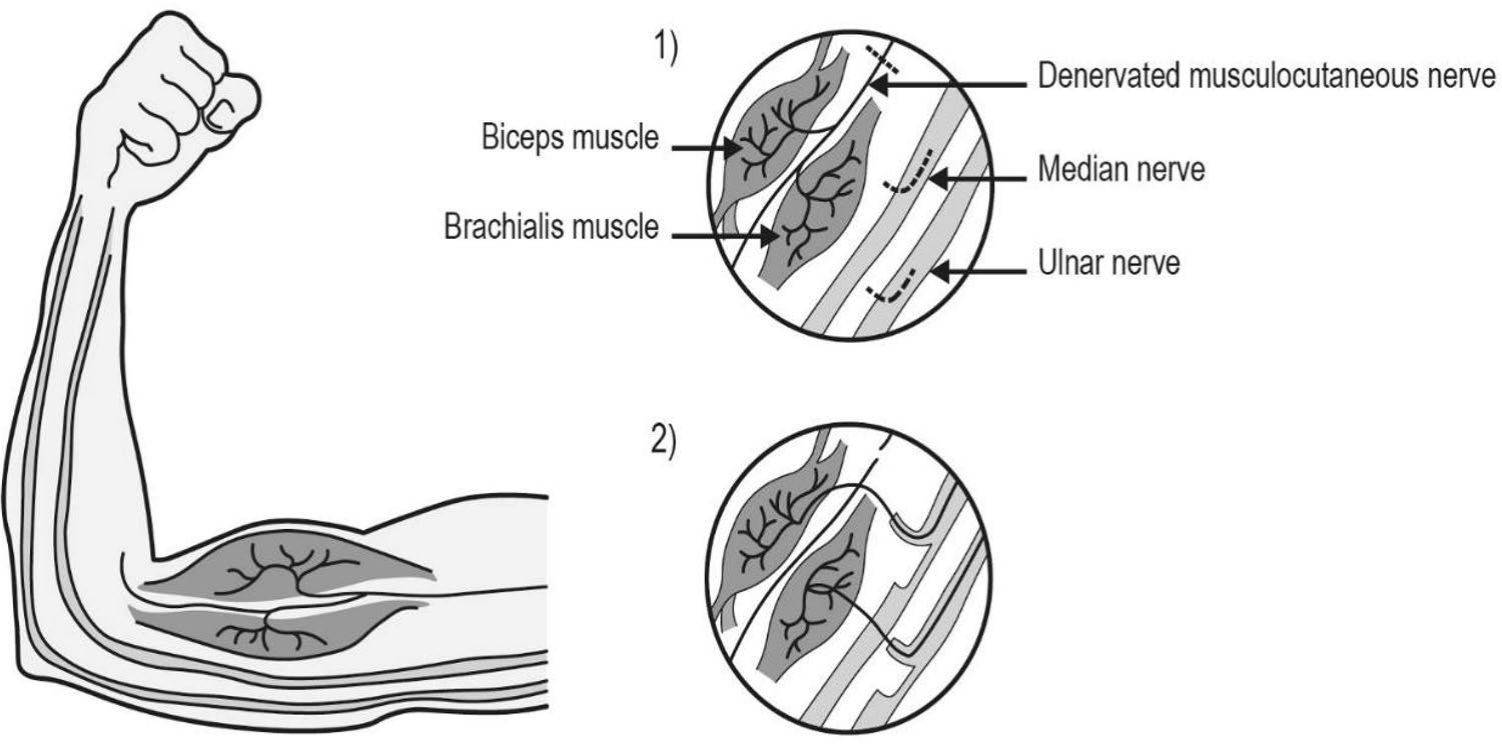
Nerve transfer to reanimate elbow flexion^43^. Restoration of elbow flexion is a common challenge encountered by the reconstructive surgeon following injury to the upper cervical roots, upper trunk, lateral cord and/or musculocutaneous nerve. The surgeon performs a neurotomy in the longitudinal orientation along donor median and ulnar nerves. Using low amplitude stimulation, a fascicle (no greater that 1/8th the size of the donor nerve) that demonstrates predominantly wrist flexor activity (flexor carpi ulnaris/flexor carpi radialis) is identified. Other fascicles are subsequently stimulated to ensure that wrist flexion would be maintained following donor harvest. Fascicles that demonstrate intrinsic hand function upon stimulation are avoided. The donor median and/or ulnar fascicles are then transferred into the chronically denervated stump of the biceps branch of the musculocutaneous nerve.

The nerve transfer procedure transects functioning fascicle(s) to allow the redirection and ingrowth of these axons into a denervated distal nerve to restore function. In this procedure to reinnervate the elbow flexors donor fascicles from the ulnar and/or median nerves are transferred into the denervated musculocutaneous nerve to brachialis and biceps (Fig. 4).

### Participant selection

**Figure 5:**
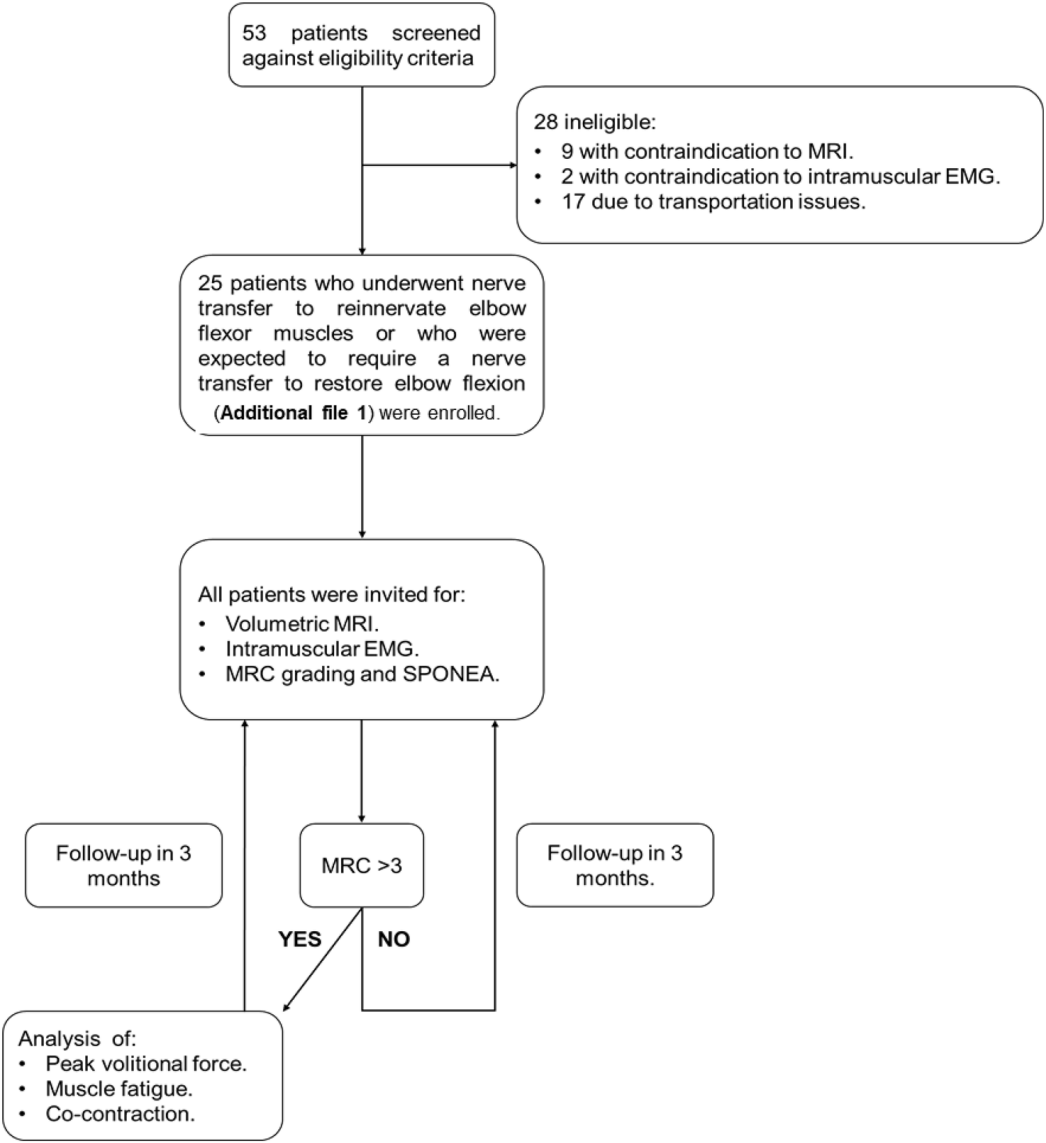
Study flow diagram. Flow diagram to illustrate study design and patient recruitment.

The study methodology identified both those who had recently undergone a nerve transfer and those due to undergo the procedure through a combined retrospective and prospective review of the institute database (Peripheral Nerve Injury Unit, Royal National Orthopaedic Hospital, UK). This process identified candidates who underwent nerve transfer to reanimate elbow flexion between August 2017 and March 2019. Patients were assessed against the following inclusion criteria: patients had to be over 18 years of age; speak fluent English; able to participate verbally with the process. Patients were excluded if they had impaired cognitive functioning or had difficulties in verbal communication and those who had suffered a birth-related brachial plexus injury. A total of 53 patients were screened against the inclusion criteria and invited to participate in the study by telephone and letter. Those who did not respond, received a follow up phone call. All patients were requested to attend MRI and neurophysiological assessment of their injured arms. Those who underwent MRI and/or neurophysiological assessment of their elbow flexor muscles were assigned an MRC grade and requested to answer the SPONEA. If patients were assigned MRC > 3 (i.e. they were able to produce force against resistance), they were also requested to participate in PVF, muscular fatigue and co-contraction assessments. Invites for additional follow-up appointments (for re-evaluation of volumetric MRI, MRC grade, intramuscular EMG and SPONEA analysis) were sent every 3 months following the previous appointment for up to 2-years post-operatively. Patients were withdrawn from the study if they no longer wished to attend additional assessments (Fig. 5).

Healthy controls were assessed against the following inclusion criteria to closely match the demographics of the study population; healthy controls had to be males aged between 18 and 60 years and must not have had any past medical history of neuromuscular disorders.

### Neurophysiology; motor unit action potential (muap) and spontaneous activity analysis

EMG analysis of size index and spontaneous activity was performed by TT. Further details on the experimental protocol used can be found in the Supplementary Material.

### MRI acquisition

#### Image acquisition

All imaging was obtained on a 3-Tesla MRI Philips scanner (Achieva, Philips, The Netherlands) using phased array coils. All patients were imaged utilising a standardised protocol with the participant in the supine position with their arm rested by their side with the palm facing upwards (Table 1). The injured arm was imaged in all 25 patients whilst the uninjured arm was imaged for comparison in six healthy male volunteers.

**Table 1:** MRI Imaging parameters.

Imaging parameter	Coronal T1-w spin echo	Coronal STIR	Sagittal T2-w fast spin echo	Axial PDW fast spin echo	Fat suppressed PDW fast spin echo
Field of view	270 × 190mm^2^	270 × 190 mm^2^	270 × 190 mm^2^	200 × 200 mm^2^	200 × 200 mm^2^
Repetition time	643	4000	3000	3000	3000
Echo time	20	80	100	30	30
Slice thickness	3 mm	3 mm	3 mm	4 mm	4 mm
Interslice gap	0.3 mm	0.3 mm	0.3 mm	0.4 mm	0.4 mm
Matrix	364 × 225	192 × 127	320 × 201	288 × 254	288 × 274
Bandwidth	361.5	1033	291.5	331	361

#### Segmentation protocol to quantify elbow flexor muscle volume

The NiftyView software (https://github.com/NifTK/NifTK) was used to perform segmentation as well as signal intensity and volume measurements. Uninjured and injured elbow flexor muscles were manually segmented using previously documented protocols which demonstrated high reproducibility and reliability^47–49^. Proximally, segmentation was commenced at the unification of the long and short heads of the biceps tendon. Once the distal part of the medial condyle of the humerus became visible, segmentation was terminated. To provide some standardisation for differences in elbow flexor muscle volume that may arise due to environmental factors, BMI measurements were obtained from participants at each MRI scan appointment. BMI has been shown to be positively correlated (R^2^ > 0.7) in a number of studies with measurements of muscle volume and upper arm diameter^50,51^. Therefore, the elbow flexor muscle volume per unit BMI was determined.

#### Scan-rescan, inter- and intra-investigator reproducibility of the segmentation protocol

One patient and three healthy volunteers underwent scan-rescan tests of their injured and healthy arms (on the dominant side) respectively to determine scan-rescan reproducibility of the MRI measurements. After the initial scan, participants were asked to get off the scanner table, rest for 5–10 min and then get back on to the table again for the second scan with the same imaging protocol. For the assessment of inter- and intra-investigator reproducibility assessment, the first 20 MRI scans to be acquired in the study were manually segmented by MW and a PhD student to quantify the ratio of elbow flexor muscle signal intensity to the signal intensity of the humeral shaft and the elbow flexor muscle volume per unit BMI acquired from T1-w, PDW and T2-w images. The Intraclass Correlation Coefficient (ICC) was used to quantify the scan-rescan, inter- and intra-investigator agreement of the segmentation protocol.

### Objective clinical assessments of muscular function

All participants were assessed using the MRC grading system of muscle power. In those determined to have MRC grade > 3, sub-analysis of PVF, muscular fatigue and co-contraction was performed (Fig. 5). Previously published protocols, optimised for the evaluation of reinnervated elbow flexor muscles, were followed^18,52,53^ (additional information provided in the Supplementary Material). All assessments were performed in the injured and uninjured, contralateral arms. Recordings were taken by MW. Peak force and/or sEMG signals were used to provide an objective appraisal of PVF, muscular fatigue and co-contraction within these assessment models. The experimental setup is shown in Supplementary Material Fig. 1.

#### Quantification of peak volitional force

PVF was recorded as the maximum force exerted by the participant during the ten repeated isometric contractions of elbow flexor muscles. This was documented for the injured and uninjured, contralateral arms.

#### Quantification of muscular fatigue

##### sEMG

MDF is a frequency value at which the EMG power spectrum is divided into two regions with an equal integrated power and is a commonly used sEMG index of muscular fatigue. The time taken for the MDF to fall by > 10% from the mean MDF obtained for the first 10 s of the sustained isometric contraction was used to estimate the physiological onset of muscular fatigue. The percentage difference in this time interval between injured and uninjured arms was quantified.

##### Force

The mean force generated in the first 10 s of the sustained isometric contraction was measured. The time taken for the mean force to drop by more than 50% of this value was determined for the injured and uninjured arms. The percentage differential between injured and uninjured arms was quantified.

##### Subjective

The time taken for patients to first report fatigue within their elbow flexor muscles during the sustained isometric contraction was recorded for injured and uninjured arms. This was performed in both the injured and uninjured, contralateral arms. The percentage difference in this time interval between injured and uninjured arms was quantified.

#### Quantification of co-contraction

The co-contraction ratio around the elbow joint was quantified using previously documented protocols^54–56^. The raw EMG signals obtained during the sustained isometric contraction of elbow flexor muscles were collected and analysed using DATALite software (Biometrics Ltd). The root mean square (RMS) of the EMG data and area under the RMS curve (AOC sEMG) was determined for elbow flexor and extensor compartments. Equation 1 was used to quantify the mean co-contraction ratio around the elbow joint for injured and uninjured arms.

1 $$Co{\rm -}contraction\,ratio=\frac{AOC\,for\,anatagonist\,muscle\,(triceps\,brachii)}{AOC\,for\,aganist\,muscle\,(biceps\,brachii)}$$

Equation (1)—Quantification of co-contraction around the elbow joint.

#### Subjective assessment of muscular function

All participants were assessed using SPONEA:

“A normal elbow is one which is pain-free, has full range of movement, normal strength and allows you to do what you feel your elbow should allow you to do. A normal elbow is scored at 100% whereas a completely useless elbow is scored at 0%. How would you rate your elbow at the present time?”

**Table:** 

0%	10%	20%	30%	40%	50%	60%	70%	80%	90%	100%

### Statistics

The responsiveness of each parameter was compared using Pearson or Spearman correlation as appropriate. Correlations were measured using the following scale of R^2^ ranges: of < 0.3 (poor), > 0.3–0.4 (weak), > 0.4–0.5 (weak to moderate), > 0.5–0.6 (moderate), > 0.6–0.7 (moderate to good), > 0.7–0.8 (good) and > 0.8 (very good). A HGP was implemented to model the recovery of muscle volume, MRC grade and SPONEA over time following surgery. Further information on how this was performed is provided in the Supplementary Material. All data are presented as mean, median and standard deviation unless otherwise stated. Two-tailed Student’s t-test and one-way ANOVA test with post-hoc Bonferroni correction was used to determine statistical significance where appropriate. A *p* value of < 0.05 was considered significant. A two-way random effects model was used to determine the ICC. ICC values are presented with 95% confidence intervals unless otherwise stated.

### Ethics approval and consent to participate

This study received ethical approval (Research Ethics Committee reference 17/YH/0413 and IRAS ID 235012 (Yorkshire and the Humber, Sheffield); IRAS ID: 202847 and Research Ethics Committee reference 17/WM/0438, IRAS ID 231428 (West Midlands and Black Country)) and all patients were recruited after fully informed written consent. Written informed consent was received from participants prior to inclusion in the study.

## Supplementary Information

Supplementary Material.

Additional File 1.

Supplementary Video 1.

## Data Availability

The datasets used and/or analysed during the current study are available from the corresponding author on reasonable request.
