# Author Correction: Volumetric MRI is a promising outcome measure of muscle reinnervation

**DOI:** 10.1038/s41598-021-03254-3

**Published:** 2021-12-06

**Authors:** Matthew Wilcox, Liane Dos Santos Canas, Rikin Hargunani, Tom Tidswell, Hazel Brown, Marc Modat, James B. Phillips, Sebastien Ourselin, Tom Quick

**Affiliations:** 1 Peripheral Nerve Injury Research Unit, Royal National Orthopaedic Hospital, Stanmore, UK; 2 UCL Centre for Nerve Engineering, University College London, London, UK; 3 Department of Pharmacology, UCL School of Pharmacy, University College London, London, UK; 4 Biomedical Engineering and Imaging Sciences, King’s College London, London, UK; 5 Department of Radiology, Royal National Orthopaedic Hospital, Stanmore, UK; 6 Department of Clinical Neurophysiology, Royal Free Hospital, London, UK; 7 University College London Medical School, London, UK

Correction to: *Scientific Reports* 10.1038/s41598-021-01342-y, published online 17 November 2021

The original version of this Article contained an error in the spelling of the author James B. Phillips which was incorrectly given as James Benjamin Phillips. The original Article and accompanying Supplementary Information file have been corrected.

