# Supplementary material for: Volumetric MRI is a promising outcome measure of muscle reinnervation
Source: Sci Rep. 2021 Nov 17;11:22433. doi: 10.1038/s41598-021-01342-y (PMC8599480; doi:10.1038/s41598-021-01342-y)
Supplement: Supplementary file 1 — Supplementary Material. [file 41598_2021_1342_MOESM1_ESM.pdf]

Volumetric MRI is a promising outcome measure of muscle reinnervation

Matthew Wilcox*[1,2,3], Liane Dos Santos Canas[4], Rikin Hargunani[5], Tom Tidswell[6], Hazel Brown[1,2], Marc Modat[4], James B. Phillips[2,3], Sebastien Ourselin[4], Tom Quick[1,2]

1) Peripheral Nerve Injury Research Unit, Royal National Orthopaedic Hospital, Stanmore, UK
2) UCL Centre for Nerve Engineering, University College London, London, UK
3) Department of Pharmacology, UCL School of Pharmacy, University College London, London, UK
4) Biomedical Engineering & Imaging Sciences, King's College London, London, UK
5) Department of Radiology, Royal National Orthopaedic Hospital, Stanmore, UK
6) Department of Clinical Neurophysiology, Royal Free Hospital, London, UK

Corresponding author: Dr. Matthew Wilcox

Institution: University College London Medical School, London, United Kingdom

e-mail: matthew.wilcox.13@ucl.ac.uk

Telephone: 07952927923

Address: Peripheral Nerve Injury Research Unit, Royal National Orthopaedic Hospital, Stanmore, HA7 4LP

Supplementary Material

Neurophysiological measurements

The Synergy UltraPro 3 Channel System was used to record MUAPs and record spontaneous activity. A concentric needle (Ambu® Neuroline Concentric 25 x 0.30 mm (1”x30G), 74025-30/25) was introduced into the biceps muscle to estimate the severity of muscle denervation from free running EMG recordings by a Consultant neurophysiologist (TT) who was blinded to the time since surgery. The following scale was used to semi-quantitatively evaluate spontaneous activity:

Severe	Moderate to severe	Moderate	Mild to moderate	Mild	None

In addition, a minimum of 30 different MUAPs were recorded from different locations (medial, mid-line and lateral) and depths of the reinnervated muscle. This process was repeated on the contralateral uninjured arm. The amplitude and duration of MUAPs was used to calculate the size-index of MUAPs (size index = 2 × $\log_{10}$(amplitude) + area/amplitude). This parameter was chosen as a measure of MUAP size since unlike other MUAP measurements it is less dependent on where the needle is placed [1,2]. In addition, this measure has demonstrated some reliability and responsiveness to the biological process of reinnervation [1-3]. The ratio of the size index recorded from the injured side to the uninjured side was calculated.

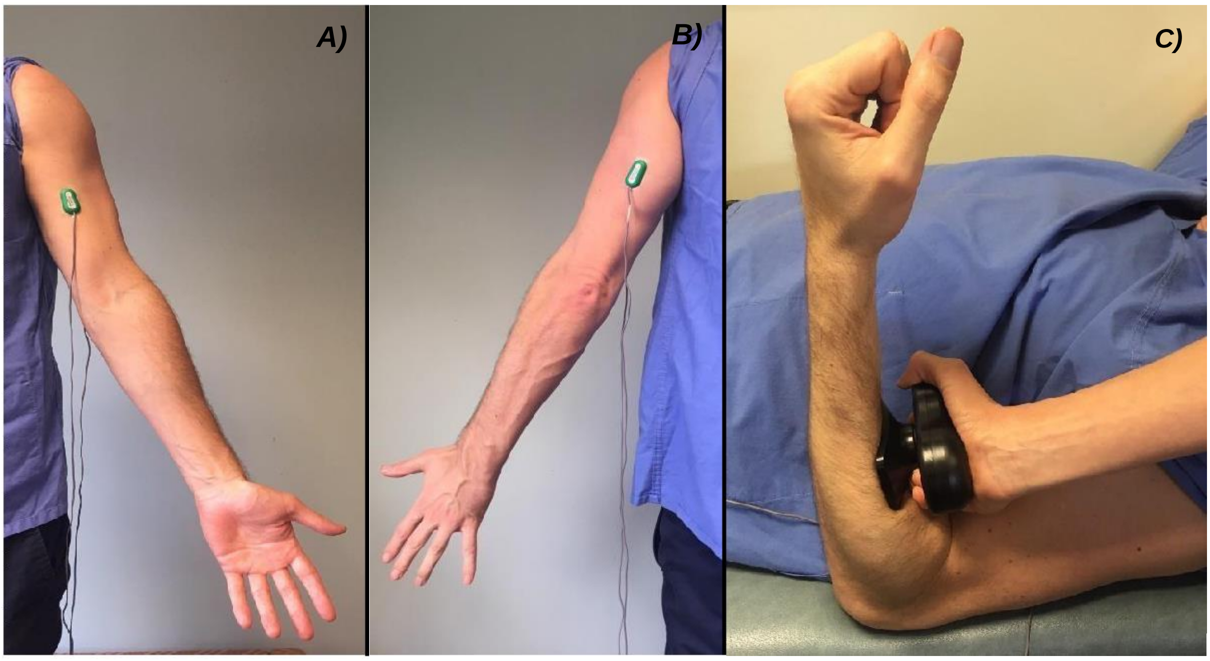

Figure 1 - A visual representation of the study setup. **A)** Placement of the recording electrode over the elbow flexor compartment (Biceps-Brachialis), **B)** Placement of the recording electrode over the elbow extensor compartment (Triceps-brachii) **C)** Positioning of the HHD for force measurements during the sustained and repeated models of fatigue assessment.

Additional experimental information concerning HHD and sEMG measurements

The plate of the HHD (M550 MyoMeter; Biometrics Ltd, Newport, United Kingdom) was applied to the forearm 10cm distal to the elbow crease with the elbow in 90º flexion and maximal supination with the fingers maximally extended. This technique aims to bias elbow flexion to biceps and brachialis [4]. Force as transduced from the HHD was continuously assesses and recorded by the DATALite software (Biometrics Ltd). sEMG signals were recorded using a four-channel surface microprocessor-controlled programmable gain amplifier (Data Log MWX8; Biometrics Ltd; 3-dB bandwidth, 10 Hz to 500 Hz), and sampled at a rate of 2048 samples per second per channel. Data were recorded using a Biometrics SX230 precision bipolar sensory monitor with a distance between electrodes of 20 mm. Prior to applying the electrode, the skin was cleaned with alcohol. The positions of the electrodes were determined in accordance with international guidelines [5]. The zone in which they were placed was standardized as one-third of the distance along a line from the acromion to the centre of

the antecubital fossa in line with the long axis of the arm for the biceps brachii and half-way along a line between the posterior acromion and the olecranon for the triceps brachii. A reference electrode was placed around the contralateral wrist (**Supplementary Material Figure 1**).

Statistical analysis

The objective of the present study was to establish the validity of volumetric MRI as an outcome measure in muscle reinnervation. In order to address this, the present study investigated three hypotheses:

1) Predictive value of muscle volume per unit BMI based on the first muscle volumetric assessment and temporal information.
2) Prediction of MRC grade based on volumetric and temporal information.
3) Prediction of SPONEA based on volumetric and temporal information.

In order to test these hypotheses, a Hierarchical Gaussian Process model (HGP) was implemented to predict the recovery of muscular function post-operatively using temporal and muscle volume per unit BMI measurements. This model consists of a Gaussian Process (GP) which allows for the inference of temporal trajectory of the biomarkers while accounting for the co-variance across subjects. The hierarchical structure is then employed to encode the individual evolution of the biomarkers, encoding the correlation between the different time-points for a same subject. As a result, the GP has been successfully deployed in a number of studies to infer the prognosis of subjects given a set of biomarkers. Unlike other time-series based approaches, GPs do not require standardised time points in the sample for analysis. Therefore, this model removes any restriction on temporal sampling and can deal with randomly and systematically missing data. Given this property, this makes the GP model useful for the dataset in the present study.

In detail, the GP model used in this study was designed to deal with specific structures in the data such as longitudinal information by including a layer of hierarchy to account for differences between subjects over time, as proposed by [6]. In addition, the structure defined by the HGP leads to the clustering of time points for each subject whilst the hierarchical layer accounts for temporal covariance of the biomarkers within that cluster.

In summary, this approach models the variance within subjects across different time points whilst modelling the sample behaviour across all subjects' outcomes for the different time points.

A)

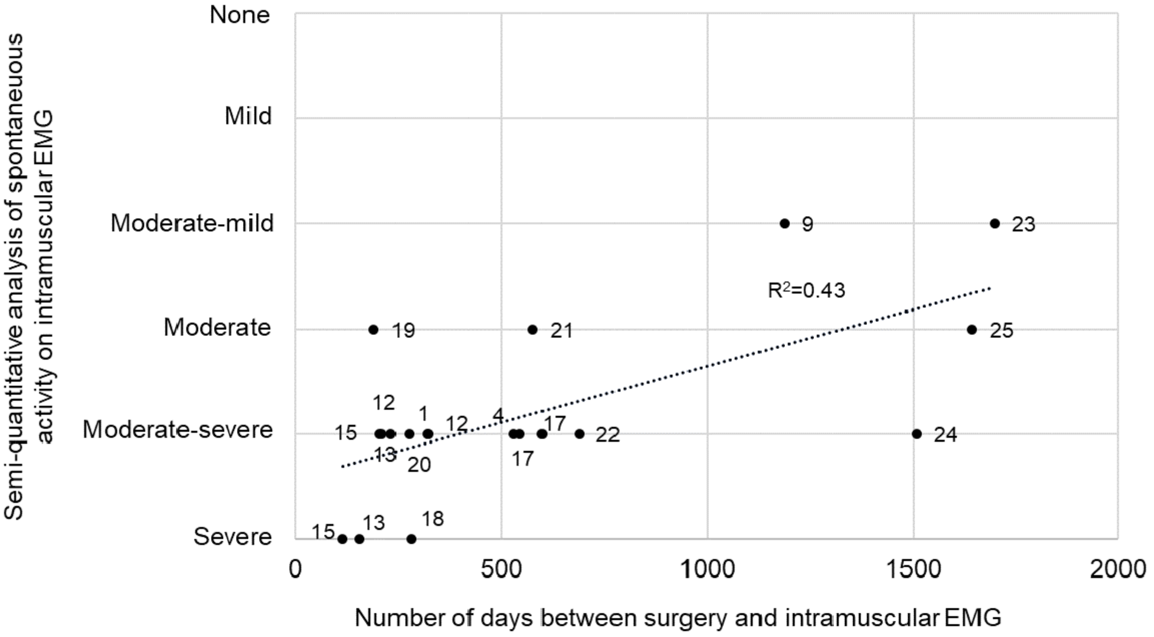

B)

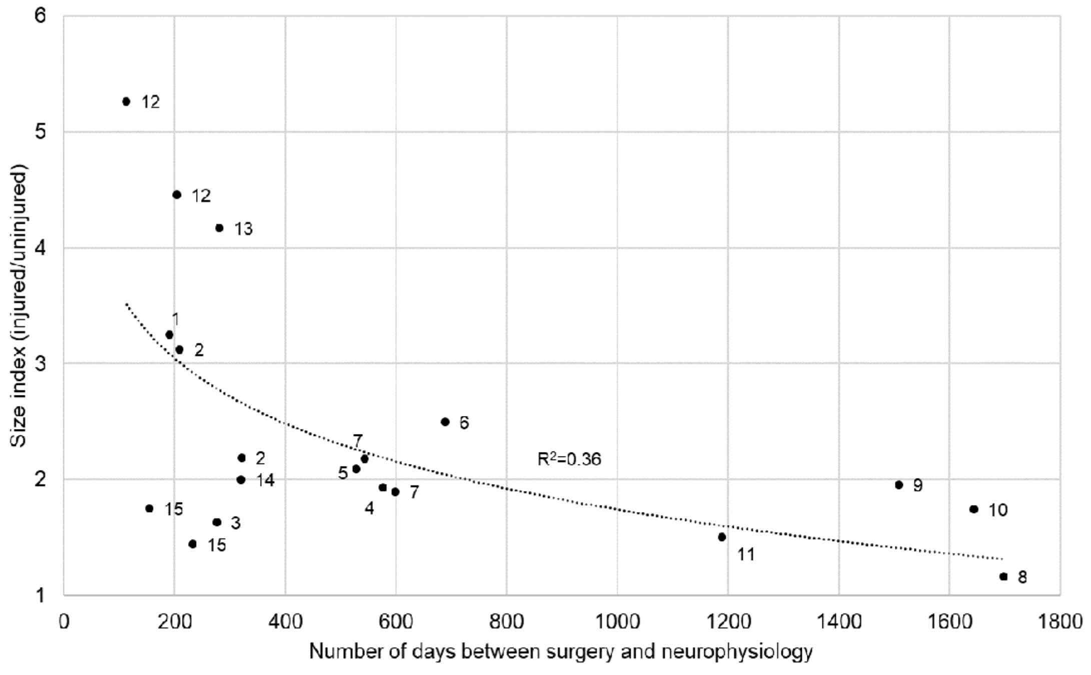

Figure 2 - Neurophysiological assessment of reinnervated muscle following nerve transfer to reanimate elbow flexion. A) Semi-quantitative assessment of spontaneous activity post-operatively. **B)** Size index quantification of MU magnitude post-operatively.

Quantitative MRI parameter	Scan-rescan reproducibility	Inter-investigator reproducibility	Intra-investigator reproducibility
T1-w volume	0.95 (0.50 - 0.93)	0.98 (0.98 - 1.00)	0.99 (0.98 - 1.00)
PDW volume	0.97 (0.72 - 1.00)	0.86 (0.63 - 0.95)	0.98 (0.95 - 0.99)
T2-w volume	0.94 (0.17 - 1.00)	0.98 (0.95 - 0.99)	0.99 (0.98 - 1.00)

Figure 3 - Scan-rescan, inter- and intra-investigator reproducibility of the imaging and segmentation protocol. Scan-rescan was performed on the dominant arm of three healthy volunteers and the injured arm from case number 6 (**Additional file 1**). Inter- and intra-investigator reproducibility was determined from the first 15 MRI scans saved to the research database (case numbers 1-12 (**Additional file 1**) and the dominant arm of three healthy volunteers). A two-way random effects model was used to determine the ICC. Values represent the ICC value with a 95% confidence interval in brackets.

	Mean absolute error (MAE)	Mean Log Likelihood	Logarithmic Error
Muscle Volume	0.896 (0.803)	0.401 (0.214)	0.036 (0.049)
MRC Grade	0.731 (0.387)	0.276 (0.061)	0.106 (0.138)
SPONEA Scale	1.696 (0.895)	0.151 (0.060)	0.502 (0.671)

Figure 4 - Predictive error associated with the predictions of the Hierarchical Gaussian Process model. Mean and standard deviation (in brackets) across subjects are presented for all subjects. These metrics are computed using a stratified leave-n-groups-outcross-validation, where 80% of the groups are used for training and the remaining is saved for testing. Note that a group consists of a set of time-points for a given subject. The mean absolute error (MAE) between the observed measurements of volume muscle, MRC and SPONEA and the predictive values are used to evaluate the performance of the model and its relevance in the clinical context. The mean logarithmic likelihood is also used as a proxy of the certainty of the model predictions.

References

1 Sonoo, M. & Stålberg, E. The ability of MUP parameters to discriminate between normal and neurogenic MUPs in concentric EMG: analysis of the MUP "thickness" and the proposal of "size index". *Electroencephalography and Clinical Neurophysiology/Evoked Potentials Section* **89**, 291-303, doi:https://doi.org/10.1016/0168-5597(93)90068-Z (1993).

2 Cercone, M. *et al.* Asymmetric recurrent laryngeal nerve conduction velocities and dorsal cricoarytenoid muscle electromyographic characteristics in clinically normal horses. *Scientific Reports* **9**, 2713, doi:10.1038/s41598-019-39189-z (2019).

3 Alix, J. J. P. *et al.* Assessment of the reliability of the motor unit size index (MUSIX) in single subject "round-robin" and multi-centre settings. *Clinical Neurophysiology* **130**, 666-674, doi:https://doi.org/10.1016/j.clinph.2019.01.020 (2019).

4 Kahn, L. C. & Moore, A. M. Donor Activation Focused Rehabilitation Approach: Maximizing Outcomes After Nerve Transfers. *Hand clinics* **32**, 263-277, doi:10.1016/j.hcl.2015.12.014 (2016).

5 Hermens, H. J., Programme, C. o. t. E. C. B. & Health, R. *European Recommendations for Surface Electromyography: Results of the SENIAM Project*. (Roessingh Research and Development, 1999).

6 Hensman, J., Lawrence, N. D. & Rattray, M. Hierarchical Bayesian modelling of gene expression time series across irregularly sampled replicates and clusters. *BMC bioinformatics* **14**, 252-252, doi:10.1186/1471-2105-14-252 (2013).
