# Supplementary material for: Volumetric MRI is a promising outcome measure of muscle reinnervation
Source: Sci Rep. 2021 Nov 17;11:22433. doi: 10.1038/s41598-021-01342-y (PMC8599480; doi:10.1038/s41598-021-01342-y)
Supplement: Supplementary file 2 — Additional File 1. [file 41598_2021_1342_MOESM2_ESM.pdf]

Patient demographics.

Case Number	Age range (Gender)	Mechanism of injury	Intra-operative findings	Operation details	Number of days between injury and surgery	Number of days between surgery and neurophysiological assessment	Number of days between surgery and MRI scan	Peak volitional force, muscle fatigue and co-contraction assessment?
1	30-39 (M)	Motorbike accident	C5-8 Avulsion	Right double Oberlin's, spinal accessory to suprascapular	15	319	28 and 213	Yes (213 days post-operatively)
2	30-39 (M)	Motorbike accident	C5/6 Avulsion	Right spinal accessory to suprascapular and right Oberlin's	173	N/A	45 and 239	No
3	20-29 (M)	Motorbike v tree	Axonotmesis of biceps branch of musculocutaneous nerve	Left Oberlin's	121	N/A	292 and 485	Yes (292 and 485 days post-operatively)
4	20-29 (M)	Motorbike v car	C5/6 Avulsion	Left spinal accessory to suprascapular and double Oberlin's	30	529	70 days pre-operatively, 47 and 136 days post-operatively	No
5	20-29 (M)	Motorbike v Car	C5/6 Avulsion	Left spinal accessory to suprascapular and Oberlin's	76	N/A	338 and 513	Yes (513 days post-operatively)
6	50-59 (M)	Motorbike accident	C5/6 Avulsion	Left Oberlin's	323	N/A	35, 87 and 580	No
7	40-49 (M)	Bicycle v Car	C5/6 Avulsion	Right double Oberlin's and Somsak's	44	N/A	539	Yes (539 days post-operatively)
8	50-59 (F)	Motorbike accident	C5/6 Avulsion	Left Oberlin's	242	N/A	193, 336 and 533	No
9	30-39 (F)	Car v Lorry	C5/6 Avulsion	Right Oberlin's	45	1189	1419	Yes (1419 days post-operatively)
10	20-29 (M)	Motorbike accident	C5/6 Avulsion	Right Oberlin's	92	N/A	1626	No
11	20-29 (M)	Bicycle v Bus	C5/6 Avulsion	Right Oberlin's and Somsak's	433	N/A	1371	Yes (1371 days post-operatively)
12	20-29 (M)	Motorbike v car	C5/6 Avulsion	Right Oberlin's and suprascapular to spinal accessory	97	209 and 321	1145	Yes (1146 days post-operatively)
13	30-39 (M)	Motorbike accident	C5/6 Avulsion	Right Oberlin's	168	155 and 232	58 and 226	No
14	30-39 (M)	Motorbike v Lamppost	C5/6 Avulsion	Left Oberlin's	356	N/A	41 and 267	No
15	30-39 (M)	Motorbike v car	C5/6 Avulsion	Left Oberlin's	143	113 and 204	86 days pre-operatively and 162 days post-operatively	No
16	40-49 (M)	Motorbike accident	C5/6 Avulsion	Left spinal accessory to suprascapular, Oberlin's and Somsak's	278	N/A	114 and 196	No
17	20-29 (M)	Motorbike accident	C5/6 Avulsion	Left spinal accessory nerve to suprascapular and Oberlin's	107	543 and 599	64 and 248	No
18	60-69 (M)	Skiing accident	C5/6 Avulsion	Left Oberlin's	141	381	223 and 388	No
19	20-29 (M)	Motorbike v Car	C5/6 Avulsion	Left Oberlin's	191	190	N/A	No
20	40-49 (M)	Motorbike v Car	C5/6 Avulsion	Right Oberlin's	97	276	N/A	No
21	30-39 (M)	Motorbike v Car	C5-C7 Avulsion	Left Oberlin's	11	577	N/A	No
22	20-29 (M)	Motorbike v Car	C5/6 Avulsion	Left Oberlin's	290	689	N/A	No
23	30-39 (M)	Motorbike v Lamppost	C5/6 Avulsion	Right Oberlin's	56	1698	N/A	No
24	20-29 (M)	Motorbike v Car	C5/6 Avulsion	Left Oberlin's	212	1509	N/A	No
25	40-49 (M)	Motorbike v Car	C5/6 Avulsion	Left Oberlin's	146	1644	N/A	No
